# Different patterns of colonization of *Oxalis alpina* in the Sky Islands of the Sonoran desert via pollen and seed flow

**DOI:** 10.1002/ece3.4096

**Published:** 2018-04-27

**Authors:** Jessica Pérez‐Alquicira, Stephen G. Weller, César A. Domínguez, Francisco E. Molina‐Freaner, Olga V. Tsyusko

**Affiliations:** ^1^ Departamento de Botánica y Zoología CONACYT – Laboratorio Nacional de Identificación y Caracterización Vegetal Centro Universitario de Ciencias Biológicas y Agropecuarias Universidad de Guadalajara Zapopan Mexico; ^2^ Department of Ecology and Evolutionary Biology University of California Irvine California; ^3^ Departamento de Ecología Evolutiva Instituto de Ecología Universidad Nacional Autónoma de México Ciudad de México México; ^4^ Departamento de Ecología de la Biodiversidad, Estación Regional del Noroeste Instituto de Ecología Universidad Nacional Autónoma de México Hermosillo México; ^5^ Department of Plant and Soil Sciences University of Kentucky Lexington Kentucky

**Keywords:** distyly, genetic structure, microsatellites, phylogeography, polyploid, tristyly

## Abstract

Historical factors such as climatic oscillations during the Pleistocene epoch have dramatically impacted species distributions. Studies of the patterns of genetic structure in angiosperm species using molecular markers with different modes of inheritance contribute to a better understanding of potential differences in colonization and patterns of gene flow via pollen and seeds. These markers may also provide insights into the evolution of reproductive systems in plants. *Oxalis alpina* is a tetraploid, herbaceous species inhabiting the Sky Island region of the southwestern United States and northern Mexico. Our main objective in this study was to analyze the influence of climatic oscillations on the genetic structure of *O. alpina* and the impact of these oscillations on the evolutionary transition from tristylous to distylous reproductive systems. We used microsatellite markers and compared our results to a previous study using chloroplast genetic markers. The phylogeographic structure inferred by both markers was different, suggesting that intrinsic characteristics including the pollination system and seed dispersal have influenced patterns of gene flow. Microsatellites exhibited low genetic structure, showed no significant association between geographic and genetic distances, and all individual genotypes were assigned to two main groups. In contrast, chloroplast markers exhibited a strong association between geographic and genetic distance, had higher levels of genetic differentiation, and were assigned to five groups. Both types of DNA markers showed evidence of a northward expansion as a consequence of climate warming occurring in the last 10,000 years. The data from both types of markers support the hypothesis for several independent transitions from tristyly to distyly.

## 1 INTRODUCTION

Climatic oscillations have dramatically impacted population size and distribution of species and thus the patterns of genetic diversity and traits that are targets of natural selection (Hewitt, 2000, 2004; Masta, 2000; Pérez‐Alquicira et al., 2010). Historical changes including climatic oscillations may have also influenced the evolution of plant reproductive systems. Analyzing the historical landscape changes combined with information derived from genealogies can contribute to a better understanding the evolutionary history of a reproductive systems of plants. Recent studies have investigated the influence of historical processes such as vicariance events related to climatic changes on the evolutionary dynamics of the plant breeding systems (Dorken & Barrett, 2004; Hodgins & Barrett, 2007; Pérez‐Alquicira et al., 2010; Zhou, Barrett, Wang, & Li, 2012). Furthermore, selective pressures associated with the immobility of plants and their reliance on pollen vectors have played an important role in the expression of breeding system variability (Barrett, 2013).

Regions with climatic oscillations that have impacted distribution of organisms represent natural laboratories for investigation of how historical processes such as bottlenecks, isolation, genetic drift, and natural selection have produced complex configurations of population structure and influenced the evolution of reproductive systems (Boyd, 2002; Masta, 2000; Sosenski, Fornoni, Molina‐Freaner, Weller, & Dominguez, 2010). An area that has experienced drastic changes in the configuration of the ecosystem is the current desert of northwestern Mexico and southwestern USA. During the last glacial maximum (18,000 years ago), the Sonoran and Arizona deserts experienced an average decrease in temperature of 6°C and increase in humidity (Metcalfe, 2006), fostering an extensive expansion of woodland vegetation. During the last 10,000 years, the climate in these areas became warmer and drier, producing a northward and upward range shift of cool‐temperate species. Currently, these species are restricted to the tops of the isolated mountains surrounded by lower elevation desert, known as the Sky Islands of the Sonoran Desert, which serve as habitat for cool‐temperate species. On these isolated peaks, founder events associated with the colonization of new areas or bottlenecks of populations that have decreased in size following the warming should produce an impoverishment of genetic variation. The genetic evidence provided by phylogeographic studies in the Sky Island region is limited (Pérez‐Alquicira et al., 2010), although a latitudinal gradient of genetic diversity as a consequence of northward migrations during Holocene period has been evident in species inhabiting the northern Mexican deserts (Clark‐Tapia & Molina‐Freaner, 2003; Cuevas, Arias, Dominiguez, Castillo, & Molina‐Freaner, 2006; Silva‐Montellano & Eguiarte, 2002). Several studies have also detected similar latitudinal gradients and western colonization during the Holocene Epoch in species inhabiting Europe (Conord et al., 2012).

**Figure 1:**
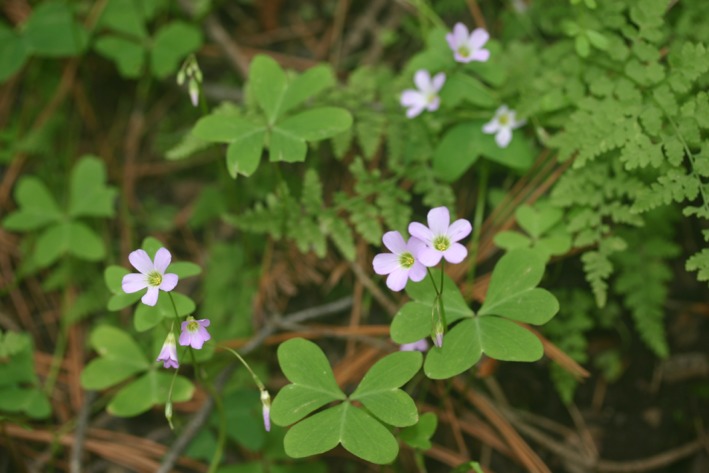
*Oxalis alpina* (Rose) Knuth growing in the Chiricahua Mts., southeastern Arizona

In order to have a general overview of the evolutionary history of population structure, molecular markers with both cytoplasmic and nuclear inheritance should be used. Because of the differences in the inheritance of nuclear and cytoplasmic genes (chloroplast and mitochondria), the patterns of genetic structure are frequently dissimilar. On one hand, the dispersion of nuclear genes occurs via pollen and seeds, while the cytoplasmic genes are maternally inherited in most angiosperms, and thus, dispersal of cytoplasmic genes depends exclusively on seed movement (Ennos, 1994; Sears, 1980). Gene flow through seeds is usually more restricted in comparison with pollen flow, and population structure should be compared using plastid and nuclear loci that are expected to differ in the magnitude of gene flow. In this study, we performed a comparison of genetic structure through cpDNA (results previously published in Pérez‐Alquicira et al., 2010) and nuclear microsatellite markers of alpine wood sorrel, *Oxalis alpina* (Figure 1).

**Figure 2:**
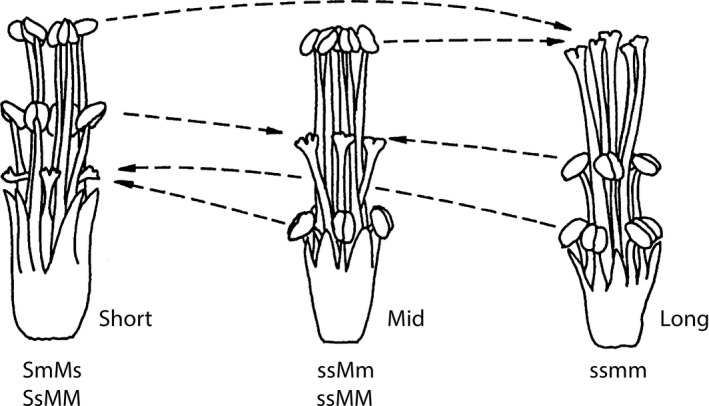
Reproductive system of *Oxalis alpina* including three floral morph and their possible genotypes. The genetic system controlling tristyly (remove coma), consists of two linked loci, each with two alleles. The S locus is epistatic over M. The presence of the dominant S allele results in the expression of short‐styled morph, while the dominant M allele produces the expression of midstyled morph, and when both loci are homozygous, the long‐styled phenotype is expressed. The dotted lines indicate the legitimate crosses, those leading to substantial seed production

*Oxalis alpina* (Rose) Knuth (section *Ionoxalis*, Oxalidaceae) is a heterostylous, tetraploid, perennial herb inhabiting evergreen Madrean woodlands in the Sky Island region (Weller, Dominguez, Molina‐Freaner, Fornoni, & LeBuhn, 2007). Heterostyly is a floral polymorphism where two (distyly) or three (tristyly) floral morphs occur in populations. Tristyly includes short‐, mid‐, and long‐styled morphs; the mid‐styled morph is absent in the distylous populations. Distyly is the derived breeding system in *Oxalis* (Gardner, Vaio, Guerra, & Emshwiller, 2012; Weller & Denton, 1976; Weller et al., 2007). Each floral morph is characterized and named by the position of the stigma relative to the two levels of stamens (Figure 2). Typically, heterostylous incompatibility systems prevent self‐fertilization and fertilization between stigmas and anthers that are located at different heights (illegitimate crosses, those crosses that do not produce seeds, sensu Darwin, 1877) (Barrett, 1992). For example, legitimate crosses occur between the short stigma and pollen from the short stamens of the long‐ and midstyled morphs. Legitimate crosses of the long‐styled morph occur when pollen comes from the long stamens of the short‐ and midstyled morphs, and the midstigma produce seeds after receiving pollen from the mid stamens of the short‐ and long‐styled morphs (Figure 2; Barrett, 1992).

*Oxalis alpina* exhibits remarkable variation in the reproductive system when viewed in the historical and geographic context of the Sky Island region. First, tristyly occurs mainly in southern ranges while the derived distylous reproductive system occurs in the northern ranges. Great variation in the frequency of the floral morphs has been observed among tristylous populations in the Sky Islands (Weller et al., 2007 ). Second, the incompatibility system includes different degrees of modification, with remarkable modifications in the northern ranges of the distribution. Weller et al. (2007) found a negative relationship between the extent of incompatibility modification in the short‐ and long‐styled morphs and the frequency of the midstyled morph, suggesting that the modifications of the incompatibility system have influenced the loss of midstyled morph in distylous populations (Weber et al., 2013). As the mid‐styledmorph becomes less frequent, floral morphology of the remaining short‐ and long‐styled morphs more closely resembles the morphological patterns of distylous populations (Sosenski et al., 2010).

Historical factors associated with range expansion and contraction during climatic oscillations in the Pleistocene Period might also have influenced the tristyly–distyly transition (Pérez‐Alquicira et al., 2010). We previously demonstrated that northern populations exhibited the lowest levels of genetic diversity suggesting this area was recently colonized by *O. alpina*. Thus, genetic drift associated with founder events in northern ranges might have influenced the loss of midmorph in populations that already have low frequencies of the midstyled morph (Pérez‐Alquicira et al., 2010). Studying the evolutionary transitions of the breeding system of *O. alpina* in the historical and geographic context of the Sky Islands provides a unique opportunity to capture the evolutionary steps in the tristyly–distyly transition. In this study, we (1) analyze the influence of climatic oscillations on the genetic structure of *O. alpina* using microsatellite markers and propose phylogeographic scenarios to aid in understanding of the evolutionary processes behind the variation in the reproductive system of *O. alpina* and (2) compare the phylogeographic patterns inferred from the microsatellite data with previous findings from cpDNA data to reconstruct the historical scenarios that better explain the current patterns of genetic structure of *O. alpina* populations.

## 2 MATERIALS AND METHODS

### 2.1 Study species

*Oxalis alpina* occurs from southwestern USA to Guatemala. Based on molecular evidence, the North American *Oxalis* section *Ionoxalis* species colonized this area in two events from ancestral southern South America species (Gardner et al., 2012). Furthermore, based on the same study, the populations of *O. alpina* from central Mexico and those in the Sky Islands are not monophyletic. In contrast, populations located in the Sky Island region are monophyletic based on morphology, ploidy levels (Weller & Denton, 1976), and their genetic relatedness (Pérez‐Alquicira et al., 2010). Pollinators are solitary bees (*Heterosarus bakeri* and *H. neomexicanus*; Andrenidae) (Baena‐Díaz et al., 2012; Weller, 1981) and sporadic visitors including other bees and various species of Diptera (Baena‐Díaz et al., 2012). Fruits are small capsules that explode when they are mature and release seeds in proximity to the plant, suggesting restricted seed dispersal.

### 2.2 Sampling

We collected bulbs from populations of *O. alpina* at the Sky Island region from 2001 to 2004. Bulbs were grown in a soil‐less mix at the University of California Irvine greenhouse. Foliar tissue (two leaves) was collected from 10–68 individuals per population (Table 1). Bulbs were collected at distances of >1 m to minimize sampling of the same genotype. The tissue was preserved in silica gel until the DNA was extracted. We sampled a total of 17 populations for microsatellite markers (Table 1).

**Table 1:** Breeding system and geographic coordinates for 17 populations of *Oxalis alpina* from the Sky Island region

Population	Pop abbrev	Mid freq	Geog. coord. (latitude; longitude)	RS
Pinaleño (954)	Pinale	0	32.6459; −109.8508	D
Santa Rita (976)	Rita	0	31.7026; −110.8683	D
Pinery Chiricahua (774)	Pinery	0	31.9328; −109.2718	D
Crest Trial Chiricahua (776)	Crest	0	31.894; −109.2819	D
Sierra Ancha (781)	Ancha	0	33.8404; −110.9556	D
Pinal (972)	Pinal	0	33.2996; −110.8415	D
Miller Canyon Huachuca (702)	Huach	0.28	31.4158; −110.2779	T
Morse Canyon Chiricahua (727)	Morse	0.25	31.8326; −109.3285	T
La Púrica (968)	Purica	0.30	30.5492; −109.7512	T
Animas (973)	Anima	0.29	31.5671; −108.7774	T
Mariquita (960)	Mariq	0.36	31.0537; −110.3834	T
Pinos Altos (971)	Altos	0.21	32.9223; −108.2126	T
Galiuro (978)	Galiur	0.20	32.5170; −110.2639	T
White (713)	White	0.26	33.6824; −109.4482	T
Buenos Aires (961)	B.aire	0.33	30.7285; −109.8343	T
Elenita (956)	Elenita	0.32	31.0461; −110.3827	T
Azul (966)	Azul	0.34	30.7412; −110.5732	T

D, distylous; RS, reproductive system; T, tristylous.

### 2.3 DNA extractions, amplifications, and microsatellite genotyping

Genomic DNA was isolated from 100 mg of leaf tissue following Doyle and Doyle CTAB procedure (1987) and also using the DNeasy Qiagen Kit. The DNA was dissolved in 50 μl of DNase/RNase‐free distilled water (Invitrogen) and quantified using a fluorometer (Hoefer Biofarmacia Dynaquant 200). A total of 617 samples were used for the microsatellite analyses. Eight polymorphic microsatellite loci (Oxa17, 25, 41, 43, 62, 81, 84, and 88) described in Tsyusko et al. (2007) were selected for the analyses. The PCR reactions were performed as described in Weber et al. (2013) in a total of 12.5 μl volume (11.5 μl + 1 μl of DNA): 1× PCR buffer, 25.0 μg/ml BSA, 0.24 μmol/L of each (forward and reverse) primer (10 μm), 2 mmol/L (for Oxa17, 62, 81, and 84) or 3 mmol/L (for Oxa25, 41, and 43) MgCl_2_, 0.15 mmol/L dNTPs, 0.5 U JumpStart Taq DNA Polymerase (Sigma), and 5–50 ng DNA template. An Applied Biosystems thermal cycler (GeneAmp PCR System 9700) and ABI PRISM 3130xl sequencer, respectively, were used for amplifications and to run microsatellite PCR reactions combined with an internal size standard Naurox (DeWoody et al., 2004). For Naurox we used fluorescently labeled with Rox size standard consisting of 13 fragments (up to 424 bp long), which was prepared in‐house following the protocol described in DeWoody et al. (2004). The plasmid DNA from pUC‐19 was used as a template for the Naurox. Genemapper version 4.0 (Applied Biosystems) was used to genotype all individuals across eight microsatellite loci. Genotypic configurations (for each of the four allele copies for tetraploids) were determined using the microsatellite DNA allele counting‐peak ratios (MAC‐PR) method (Esselink, Nybom, & Vosman, 2004). The allele dosage in this method is inferred based on the ratio of the allele peak areas from the microsatellite electropherograms for each individual at every locus. We estimated the average frequency of null alleles in 17 populations of *O. alpina* based on De Silva method (De Silva, Hall, Rikkerink, McNeilage, & Fraser, 2005) implemented in POLYSAT (Clark & Jasieniuk, 2011). We found average null allele frequencies of 0.16, 0.02, 0.001, 0.02, 0.13, 0.10, 0.09, and 0.02 for loci Oxa17, 25, 41, 43, 63, 81, 84, and 88, respectively. The average population selfing rate of 0.2, as estimated in our previous study (Weber et al., 2013), was used when calculating the null allele frequencies. Given that null allele frequencies did not exceed 0.2 with an overall multilocus average of 0.07, all loci were included in the data analysis (Oddou‐Muratorio, Vendramin, Buiteveld, & Fady, 2009).

### 2.4 Data analyses

#### 2.4.1 Genetic diversity

We used GenoDive (Meirmans & Van Tienderen, 2004) to estimate clonal diversity for each population, and only genets were included in the subsequent analyses. Spatial Pattern Analysis of Genetic Diversity (SPAGeDi) version 1.3a (Hardy & Vekemans, 2002), which allows use of codominant polyploid data, was used to calculate genetic diversity parameters, including observed heterozygosity (*H*_O_), Nei's gene diversity corrected for sample size (expected heterozygosity; *H*_E_), number of alleles per locus (*N*_A_), and effective number of alleles per locus (*N*_Ae_). SPAGeDi assumes polysomic inheritance, which is expected for low‐order autopolyploids such as *Oxalis alpina*. Average allelic richness (AR) was calculated for each population using the rarefaction method implemented in Allelic Diversity Analyzer (ADZE) while limiting sample size to 20 (Szpiech, Jakobsson, & Rosenberg, 2008). We evaluated the relationship of geographic factors (latitude and longitude) with measures of genetic diversity. We performed a covariance analysis including the latitude, longitude, and the interaction of both variables; the response variables included levels of diversity through microsatellites markers (*H*_E_, *H*_O_, *N*_A_, *N*_Ae_) and cpDNA (haplotype diversity). The analyses were carried out using the program JMP (version 9.0; SAS Institute Inc., 2005).

#### 2.4.2 Genetic structure

The levels of genetic differentiation among populations measured using the parameters *F*_ST_ and *R*_ST_ were estimated using SpaGeDi software version 1.3a (Hardy & Vekemans, 2002). We also obtained the *F*_ST_ values for distylous and tristylous populations and compared our results with cpDNA data previously published (Pérez‐Alquicira et al., 2010). To test for phylogeographic pattern, we used the approach proposed by Hardy, Charbonnel, Fréville, and Heuertz (2003), which is based on the comparison of the observed *R*_ST_ (before randomization) and an expected value (*pR*_ST_) calculated after 5,000 allele size permutations using SPAGeDi version 1.3a (Hardy & Vekemans, 2002). This test can be interpreted as testing whether *F*_ST_ = *R*_ST_. If *R*_ST_ is significantly larger than *pR*_ST,_ a stepwise mutation model is the most likely explanation for genetic differentiation (Hardy & Vekemans, 2002) and a phylogeographic pattern is suggested. If the difference between *R*_ST_ and *pR*_ST_ is not significant, this suggests that allele size is not as important because mutations do not follow a stepwise mutation model, the absence of phylogeographic pattern can be inferred, and *F*_ST_ should be used instead of *R*_ST_.

To determine how many distinct population clusters were supported by the data, we used the software STRUCTURE 2.3.4 (Pritchard, Stephens, & Donnelly, 2000). This program uses a Bayesian method to determine the probability for each individual to be assigned to a particular cluster regardless of its geographic location. We ran the analyses using the admixture model and correlated frequencies with a burn‐in and run length of 250,000 and 1,000,000, respectively, *k* = 1 to 10 (*k* indicates the number of genetic clusters), and 10 iterations for each k. Further analyses of the substructure within each main genetic cluster were analyzed using the same parameters as for the entire data set (burn‐in and run length of 250,000 and 1,000,000, respectively, *k* = 1 to 10, and 10 iterations for each *k*).

We did not include the population origin as a “prior” for the analyses. The results were imported into Structure Harvester (Earl & vonHoldt, 2012), which allows the assessment and visualization of the likelihoods for each k value and detects the number of genetic clusters that best fit the data, based on Delta K (Evanno, Regnaut, & Goudet, 2005).

We further carried out a principal coordinate analysis (PCoA) using the Bruvo distances for visualizing the genetic structure and relationships among samples through the R program POLYSAT (Clark & Jasieniuk, 2011). A neighbor‐joining (NJ) phenogram was constructed based on Nei's genetic distances to visualize the genetic relationship among populations. We obtained the allelic frequencies through the program POLYSAT (Clark & Jasieniuk, 2011) and then generated 1,000 replicates by a bootstrap resampling method using the seqboot program included in the Phylip package version 3.695 (Felsenstein, 1989). Nei's genetic distances (1972) were estimated through Gendist (Phylip package version 3.695), and then, the NJ phenogram was constructed by the Neighbor package (Phylip package version 3.695). Isolation by distance was evaluated with the Mantel test (Mantel, 1967) using the package ADE4 (Dray & Dufour, 2007) in RStudio (RStudio Team, 2015). A geographic distance matrix was obtained using the program Geographical Distance Matrix Generator version 1.2.3 (Ersts,Query 11. Insert 2017^2017^, Internet) and Nei's genetic distances (1972). We constructed a population graph network described by Dyer and Nason (2004) using the popgraph package (Dyer, 2014) in R 2.15.3 (R Development Core Team 2013). The popgraph program uses a graphical theoretical approach without a priori assumptions about population structure. The method is based on the genetic covariance structure among populations analyzed simultaneously (Dyer & Nason, 2004). Populations that exhibit significant genetic matrix correlation will be connected in the network by edges (lines), and the length of the edges is inversely proportional to the genetic covariance between the populations. Therefore, longer edges indicate lower genetic covariance between populations. Populations that are not connected indicate the absence of migration, and the presence of subgraphs (a smaller network within a large network) indicates that a population or group of populations maintain a weak or null genetic connection (Dyer, 2007; Dyer & Nason, 2004; Dyer, Nason, & Garrick, 2010). In addition, we tested for a correlation between the matrices of genetic distances (Nei, 1972) and the midmorph frequencies using a Mantel test (ADE4 package Dray & Dufour, 2007, in R). The latter test was conducted to determine whether populations with a reduced or zero (distylous population) frequency of the mid morph are genetically more similar. A significant correlation would suggest that distyly evolved from the same tristylous ancestors. The mid morph frequency matrix was constructed using the program Phylip version 3.695 (Felsenstein, 1989).

#### 2.4.3 Comparison of genetic structure between cpDNA and microsatellite markers

Because one of our objectives was to compare patterns of genetic diversity using nuclear and chloroplast markers, we performed regression analyses between haplotype diversity from cpDNA (chloroplast DNA) (Pérez‐Alquicira et al., 2010) and the genetic parameters calculated from microsatellites markers for 17 populations. We further carried out a Mantel test to examine the correlation between the *F*_ST_ genetic matrices obtained from microsatellites and cpDNA sequences. We used the *F*_ST_ parameter to have a measure comparable to previously published cpDNA sequences (Pérez‐Alquicira et al., 2010).

We also estimated the pollen‐seed migration ratio (*r *= mp/ms, where mp and ms are migration values for pollen and seeds, respectively) based on equations from Ennos (1994) and further modified by Petit et al. (2005):

$$r=\frac{\left[\left(\frac{1}{G_{\mathrm{STb}}}-1\right)\left(1+F_{\mathrm{IS}}\right)-2\left(\frac{1}{G_{\mathrm{STm}}}-1\right)\right]}{\left(\frac{1}{G_{\mathrm{STm}}}-1\right)}$$

*G*_ST_ corresponds to the genetic differentiation statistic; the subindices b and m correspond to biparental and maternal inheritance markers, respectively, and *F*_IS_ corresponds to the heterozygote deficit estimated with nuclear codominant markers. Both *G*_ST_ and *F*_IS_ were estimated using the SpaGeDi software version 1.3a (Hardy & Vekemans, 2002). Overall, larger values for r indicate that gene flow by pollen is quantitatively more important than gene flow via seeds.

## 3 RESULTS

### 3.1 Genetic variation

Testing for clonal structure revealed the presence of 17 clones distributed in eight populations with the number of plants per clone varying from 2 to 5. For further analyses, we included only genets (one genotype per clone), and therefore, a total of 29 samples were removed from the analyses. The ratio of the number of genets to the number of ramets was from 0.73 to 0.99 (Table 2). Two distylous (Santa Rita and Sierra Ancha) and one tristylous (Buenos Aires) populations had the highest number of clones (exceeding 20%). Average genetic diversity estimates based on eight microsatellite loci for genets from 17 populations of *O. alpina* were *H*_O_ = 0.62, *H*_E_ = 0.61, *N*_A_ = 4.85, and *N*_Ae_ = 2.79. The total number of alleles per locus varied from 8 to 25 with an average of 15 alleles. Average allelic richness per population was 3.03 and 3.05 when taking into account biases for low sample size. In general, the lowest levels of diversity were found in the two most northern ranges, and the Sierra Ancha and White populations, however, both populations have the lowest samples size (*n* = 10 and 9, respectively). After the Sierra Ancha and White populations, the northern Pinal population exhibited the lowest levels of diversity for *H*_E_, while for the parameter *N*_Ae,_ the lowest level of diversity was found in the northern Pinaleño population; the lowest values for *N*_A_ were found in Elenita and Buenos Aires, and for *H*_O,_ the lowest value was found in Morse Canyon Chiricahua population. In contrast, the highest levels of genetic diversity for the *H*o, *H*_E_ parameter, and *N*_AE_ parameters were found in the southwestern Santa Rita population, while Pinos Altos and Mariquita exhibited the highest level of diversity for the *N*_A_ estimator (Table 2). Based on allelic richness estimates, when the sample size was standardized to 20 for all populations (excluding three populations with sizes below 20), the lowest allelic richness was found for the southern Elenita population and the northern Pinal population while Pinos Altos had the highest value. Covariance analyses showed a negative and marginally significant (*p* = .051) relationship of latitude with the levels of genetic diversity estimated by *H*_E_, based on microsatellites markers (Table 3). For cpDNA sequences, latitude had a significant effect (*p* = .03) on haplotype diversity. For both genetic markers, longitude and the interaction of latitude and longitude did not show any significant effect (Table 3). The remaining parameters of genetic diversity for microsatellites did not show any significant association with latitude and longitude.

**Table 2:** Genetic and clonal diversity characteristics, and bayesian assignment (clusters I and II) based on microsatellite genetic markers for 17 populations of *Oxalis alpina* from the Sky Island region

Population	*N*	Ng/Nr	*H*_o_	*H*_E_	*N*_A_	*N*_Ae_	AR	AR (ss20)	Cluster
Pinaleño (954)	31	1.00	0.58	0.57	5.00	2.41	4.02	2.90	II
Santa Rita (976)	31	0.73	0.72	0.69	4.63	3.59	4.05	3.34	I
Pinery Chiricahua (774)	46	0.96	0.66	0.65	5.88	3.05	4.51	3.30	I
Crest Trial Chiricahua (776)	44	1.00	0.64	0.65	5.75	3.06	4.69	3.43	I
Sierra Ancha (781)	7	0.78	0.54	0.54	3.00	2.52	2.95	2.46	II
Pinal (972)	21	1.00	0.59	0.56	4.50	2.47	3.59	2.79	II
Miller Canyon Huachuca (702)	14	0.93	0.67	0.65	5.00	2.96	4.32	3.18	II
Morse Canyon Chiricahua (727)	67	1.00	0.55	0.58	5.50	2.50	3.99	2.95	I
La Púrica (968)	46	1.00	0.67	0.62	4.25	2.69	3.53	2.88	II
Animas (973)	36	1.00	0.62	0.62	5.50	2.75	4.25	3.10	II
Mariquita (960)	66	1.00	0.58	0.64	6.38	2.92	4.31	3.21	I
Pinos Altos (971)	67	0.99	0.66	0.65	6.38	3.04	4.37	3.89	I
Galiuro (978)	23	1.00	0.67	0.64	4.50	2.91	3.94	3.05	II
White (713)	10	1.00	0.55	0.52	3.13	2.32	3.10	2.59	II
Buenos Aires (961)	26	0.76	0.62	0.61	3.75	2.64	3.49	2.89	II
Elenita (956)	20	0.87	0.61	0.57	3.75	2.49	3.23	2.59	II
Azul (966)	33	0.97	0.68	0.67	5.63	3.27	4.49	3.31	II
Total/average	588	0.94	0.62	0.61	4.85	2.79	3.93	3.05	

AR, allelic richness; *H*_o_, observed heterozygosity; *H*_E_, expected heterozygosity; *N*, sample size; *N*_A_, allele number; *N*_Ae_, effective allele number; Ng/Nr, correspond to the ratio of number of genets and ramets; ss, set sample size for the rarefaction method.

**Table 3:** ANCOVA of the effects of latitude and longitude on the patterns of genetic diversity (a) based on DNA chloroplast sequences (Pérez‐Alquicira et al., 2010) and (b) based on microsatellites markers

Source of variation	*df*	SS	*F*	*p*
(a) Haplotype diversity				
Latitude	1	0.313	5.37	.034^a^
Longitude	1	0.131	2.24	.150
Lat × Long	1	0.095	1.64	.220
Error	16	0.932		
(b) Expected heterozygosity				
Latitude	1	0.007	4.59	.051
Longitude	1	0.000	0.01	.990
Lat × Long	1	0.001	1.11	.309
Error	13	0.022		

Only parameters resulting in a marginal or significant effect are shown.

a Significance (*p* < .05).

### 3.2 Genetic structure and genetic relationship among populations

**Figure 3:**
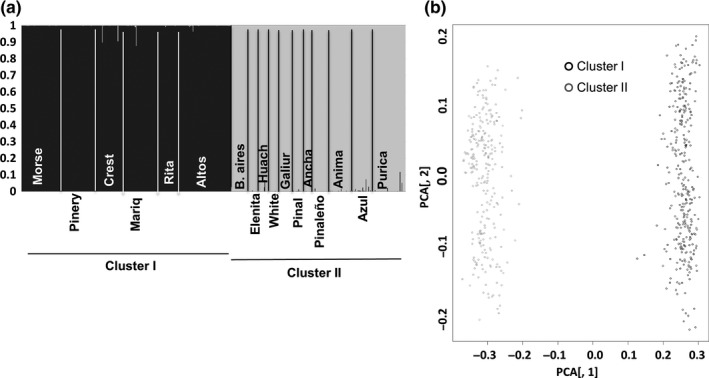
(a) STRUCTURE plot for the assignment of the samples from 17 populations of *Oxalis alpina* based on their multilocus microsatellite genotypes. (b) Principal coordinate analysis using Bruvo distances for first and second axes for 17 populations of *O. alpina* from the Sky Island region

**Figure 4:**
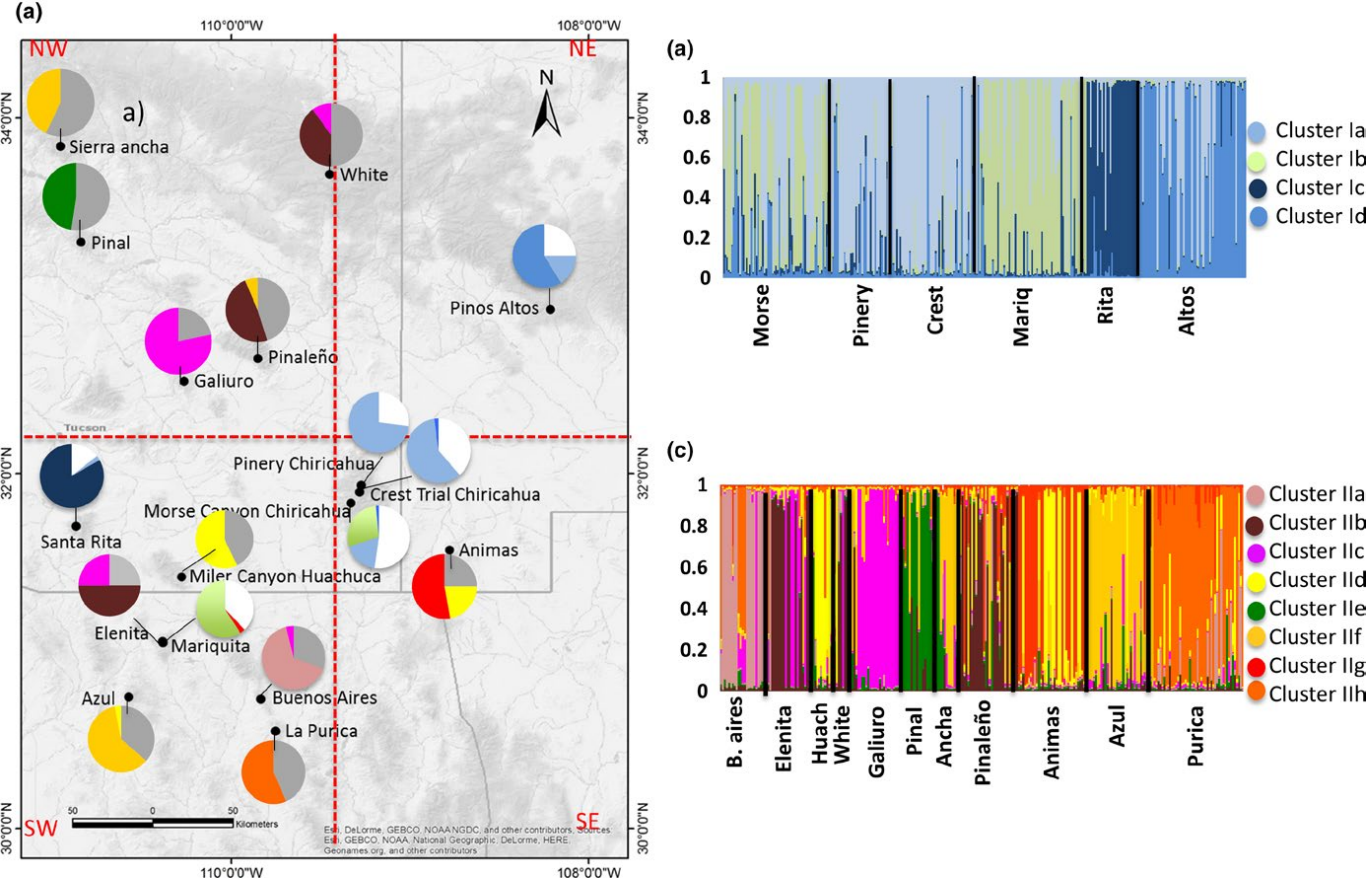
(a) Geographic distribution of the clusters generated by the Bayesian assignment implemented in the software STRUCTURE for 17 populations of *Oxalis alpina* from the Sky Island region. The pie graphs with white horizontal bars correspond to cluster I (groups Ia, Ib, Ic, and Id), while pie graphs without white bars correspond to the cluster II (IIa, IIb, IIc, and IId). Samples that were not assigned to any of the clusters because of their overlapping confidence intervals for the assignment correspond to white areas (cluster I) and gray areas (cluster II). (b) STRUCTURE plot for cluster I including six populations of *O. alpina* and (c) STRUCTURE plot for cluster II including eleven populations of *O. alpina*

We found *F*_ST_ = 0.27 and *R*_ST_ = 0.47 for 17 populations of *O. alpina*. The genetic structure for distylous and tristylous populations was similar (*F*_ST_ = 0.27 and 0.28, respectively). The *R*_ST_ parameter was marginally significantly higher than the *pR*_ST_ (0.28) (*p *=* *.053). The Bayesian individual assignments using STRUCTURE indicated that the most informative representation of the genetic structure of *O. alpina* corresponded to two groups (Figure 3a). The population samples were assigned unequivocally to either group, as the confidence intervals for the maximum probability of membership in a group did not overlap in any case. One of the groups (hereafter termed cluster I) included populations located in the northeastern (Pinos Altos), southeastern (all three Chiricahua populations), and southwestern (Santa Rita and Mariquita) quadrants of our study area. The larger group, cluster II, was composed of populations occurring throughout the entire area, with a higher incidence of populations in the southwestern area (Miller Canyon Huachuca, Elenita, Azul, Buenos Aires and La Purica populations) and populations from the northwestern area (Sierra Ancha, Pinal, Pinaleño, Galiuro, and White); and only one population from the eastern region (Animas) (Figures 3a and 4a). The PCoA analysis reflects also the presence of two main clusters, supporting the Bayesian assignment result (Figure 3b). Principal components 1 and 2 were used to construct the PCoA plot because they represent most of the total variation in the genetic data (55% and 6%, respectively). The *F*_ST_ values for clusters I and II were similar, 0.09 and 0.10, respectively, and for the parameter *R*_ST,_ the values were 0.09 and 0.059, respectively.

We also examined the genetic structure within each cluster (Figure 4). Four groups were present in cluster I (Figure 4b). Approximately 35% of the samples could not be assigned to any group, which indicates the mixed genetic nature of a large number of individuals, while 26%, 17%, 8%, and 13% of the samples were assigned to clusters Ia, Ib, Ic, and Id, respectively (Figure 4). Cluster Ia included mainly samples from the east area of the Sky Islands including the three Chiricahua populations and Pinos Altos, the Ib cluster was composed of samples from Morse Canyon (Chiricahua Mts.) and Mariquita, and the Ic cluster included only samples from the western Santa Rita population, and Id cluster was composed mainly of samples from Pinos Altos (Figure 4b). For cluster II, the maximum Bayesian probability indicated the presence of eight groups; approximately 37% of the samples could not be assigned to any group, and 6%, 11%, 9%, 6%, 4%, 9%, 7%, and 10% of the samples corresponded to clusters IIa, IIb, IIc, IId, IIe, IIf, IIg, and IIh, respectively (Figure 4c). The IIa cluster included individuals only from Buenos Aires, cluster IIb, IIc, IIf were composed of samples along the northwestern and southwestern areas of the Sky Islands, and cluster IId included samples mainly located in the southern ranges of the Sky Islands, while clusters IIe, IIg, and IIh were restricted to Pinal, Animas, and Purica, respectively (Figure 4).

**Figure 5:**
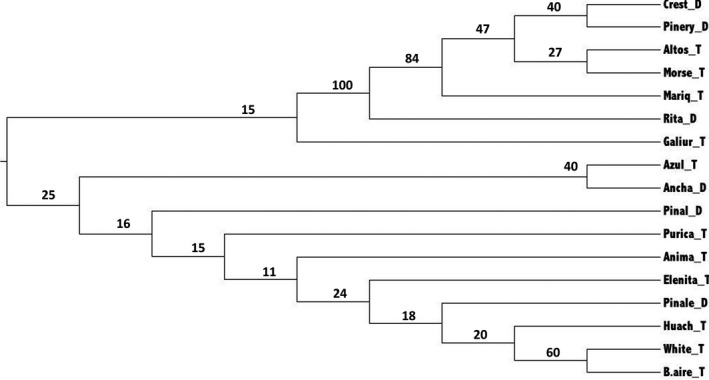
Neighbor‐joining phenogram of 17 populations of *Oxalis alpina* from the Sky Island region based on Nei's genetic distances calculated from multilocus microsatellite data. The bootstrap support is labeled for each branch

**Figure 6:**
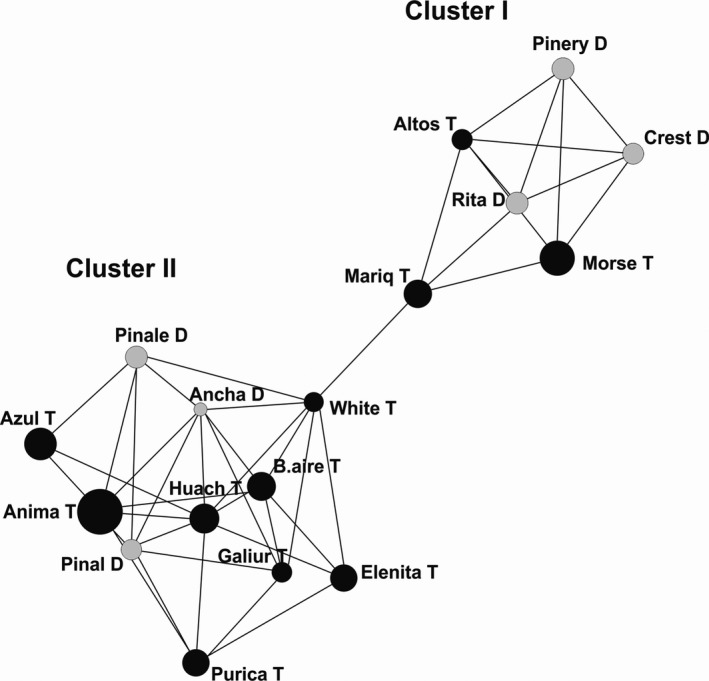
Population graph for 17 populations of *Oxalis alpina* based on nuclear microsatellites. The size of the nodes (spheres) represents the genetic variation within populations and edges (lines) connect directly two populations showing significant genetic covariance. The gray nodes correspond to distylous populations and black to tristylous populations

The neighbor‐joining phenogram recovered the Chiricahua cluster and the other three populations from cluster I with bootstrap values above 50. The remained clades were supported by very low bootstrap values (Figure 5). The Mantel test of the microsatellite data did not reveal any significant association between geographic and genetic distances (*r* = −.04, *p* = .61). The popgraph software produced a population network with two subgraphs. Each subgraph included the same populations as the two clusters (I and II) detected by the Bayesian assignment (Figure 6). Cluster I included three distylous and three tristylous populations, and 13 edges were detected. Santa Rita exhibited the highest number of connections, and it was connected with all the populations within the cluster I. The distylous Pinery and Crest populations were connected by gene flow with the tristylous Morse and Pinos Altos. The tristylous Mariquita population was the only population directly connected to the larger subgraph (cluster II) through the tristylous White population (Figure 6). The larger subgraph (cluster II) included three distylous and eight tristylous populations and 32 edges. Overall, the population network exhibited high genetic connection among some distylous and tristylous populations where each distylous population was connected to at least two tristylous ones. For example, within cluster II, the distylous population Sierra Ancha was connected to the tristylous White, Buenos Aires, Galiuro, Huachuca, and Animas populations. Additionally, the distylous Pinal and Pinaleño were connected to other tristylous populations. Huachuca exhibited the highest number of connections (8) while Azul included the lowest number of connections (3). Finally, the correlation between pairwise genetic distances and the frequency of the midmorph was not statistically significant (*r* = .03, *p* = .18).

### 3.3 Comparison of genetic structure between cpDNA and microsatellites markers

Regression analyses did not show any relationship between the microsatellite diversity parameters and cpDNA haplotype diversity (*H*_O_, *R* ^2^ = −.06, *F*_1,14_ = 0.03, *p *=* *.85; *H*_E_, *R* ^2^ = −.06, *F*_1,14_ = 0.10, *p *=* *.74; *N*_A_, *R* ^2^ = −.06, *F*_1,14_ = 0.09, *p *=* *.75; *N*_AE_, *R* ^2^ = −.07, *F*_1,14_ = 0.0, *p *=* *.92). We also performed a Mantel test to compare the similarities of genetic distance matrices (based on *F*_ST_) calculated from microsatellite and chloroplast sequence data. The correlation was not statistically significant (microsatellites and chloroplast: *r = *−.04, *p =* .62). The value obtained for the pollen‐seed migration ratio was *r *=* *5.62; we used *G*_ST_ = 0.73 for cpDNA, and *G*_ST_ = 0.26 and *F*_IS_ = −0.0064 for microsatellite markers to calculate *r*.

## 4 DISCUSSION

For most angiosperms, nuclear genes are inherited paternally via pollen and maternally via seeds, while cytoplasmic genes found in the chloroplast and mitochondria are maternally inherited (Petit, Kremer, & Wagner, 1993). Complex configurations of gene flow and differences in the distribution of genetic variability within and among populations are expected through nuclear and chloroplast markers (Petit et al., 2005). Evidence from several species of angiosperms and gymnosperms support this prediction (Petit et al., 2005). Our main results demonstrate that for *O. alpina* populations from the Sky Island region, there are differences in the patterns of genetic structure inferred from nuclear microsatellite versus cpDNA markers. Our previous phylogeographic studies of *O. alpina* (Pérez‐Alquicira et al., 2010) using cpDNA markers showed strongly reduced genetic diversity in the most northern ranges of the Sky Islands. The present study partially supports this trend because we detected the lowest levels of diversity in four northern and two southern populations. The ANCOVA analysis of microsatellite data showed a marginally significant effect of latitude on the *H*_E_ parameter, suggesting that northern migrations have left a weaker signature on the patterns of diversity on the nuclear genome, in contrast to the chloroplast genome. This pattern is probably the consequence of the lower effective population size of cytoplasmic genes in comparison with nuclear genes (Birky, Fuerst, & Maruyama, 1989; Palumbi, Cipriano, & Hare, 2001).

We found significant genetic structure for *O. alpina* in the Sky Island region; the value for *R*_ST_ was higher than *F*_ST_. According to simulations performed by Slatkin (1995), unbiased and larger values of *R*_ST_ are expected when coalescent time is longer and gene flow is low. This result is partially in accordance with the marginally significant higher value of *R*_ST_ when compared to *pR*_ST_ (similar to *F*_ST_), which suggests that the stepwise mutation model could be more suitable to explain the genetic differentiation among populations than gene flow. We should not interpret this result as evidence for phylogeographical pattern (genetically similar alleles are closer geographically) because the result is only marginally significant. *Oxalis alpina* harbors two main genetic lineages, cluster I and cluster II, suggesting a latitudinal pattern of genetic structure. The negative effect of latitude on genetic diversity was detected from the covariance analysis.

Within each cluster, the genetic structure was weak and a large number of samples were not assigned to any genetic group, suggesting a recent admixture processes within each cluster. For cluster I, an east–west geographic component was revealed. For example, cluster Ia and Id included populations located in the eastern region, while the cluster Ic corresponded to the samples from the western Santa Rita population, suggesting that this population has been isolated for a longer period of time. The genetic and geographic patterns found for cluster I are also supported by the Pleistocene distribution (18,000 years ago) predicted by niche modeling (Pérez‐Alquicira et al., 2010). The niche model for *O. alpina* was built using the GARP algorithm (Genetic Algorithm for Rule‐set Production) (Stockwell & Peters, 1999), and the results indicated that during the LGM (Last Glacial Maximum, 18,000 years ago), *O. alpina* was distributed mainly at lower elevations of the southeastern portion of the Sky Island region. Therefore, northwestern migrations occurred once the climate becomes warmer. Similar patterns of migration have been detected for plant and animal species inhabiting southern Europe and Northern Africa at the end of the Pleistocene period (Davis & Shaw, 2001; Petit et al., 2003; Schmitt, 2007). In contrast to the Santa Rita population, samples from the Morse Canyon in the Chiricahua Mts. population were assigned to different genetic groups that were shared with eastern and western populations, indicating that this area probably belongs to a region where intermixing was common.

Cluster II included the remaining eleven populations distributed across the whole range of the Sky Islands. This pattern of structure suggests that northern populations were founded by different southern lineages. For example, individuals from the Azul population probably colonized the Sierra Ancha Mts, while the remaining northern populations probably were founded by different southern lineages. Other phylogeographic studies demonstrate a corridor of vegetation from southern to northern areas in this region (Barber, 1999; Downie, 2004; Masta, 2000). Overall, the phylogeographic pattern found in *O. alpina,* where two differentiated groups were identified, is similar to the Sky Island endemic beetle *Scaphinotus petersi*, where two main clades were detected (Ober & Cannolly, 2015; Ober, Matthews, Ferrieri, & Kuhn, 2011) including a western clade with a northern–southern pattern (Huachuca, Santa Catalina, and Pinal Mts.) and a second clade located in the eastern area (Chiricahua, Pinaleño, and the western Santa Rita populations).

Alternatively, the phylogeographic pattern of strong similarities between southern–northern populations such as those observed between the northern distylous Sierra Ancha with the southern tristylous Azul populations could be explained as the consequence of homoplasy where two alleles are identical via convergence. However, because eight microsatellite loci were included in our analyses, convergence seems unlikely to explain all of these similarities. For example, Navascués and Emerson (2005) performed simulations to understand the potential effects of homoplasy on determining genetic relationships among populations. They found that as the number of loci in a study increases, the bias for the estimation of genetic parameters as a consequence of homoplasy decreases. The simulations indicated that four loci produced higher values for the homoplasy index and greater bias for inferring genetic relationship among populations than simulations using nine loci. We included eight loci in our study, and thus, bias in the inferring phylogeographic structure via homoplasy is unlikely but not impossible.

Because dispersion by pollen frequently occurs over larger distances than by seeds, lower levels of genetic structure were expected for nuclear genes in comparison with cytoplasmic genes (Birky et al., 1989; Petit et al., 1993, 2005), a pattern supported by our results for *O. alpina*. The genetic structure of *O. alpina* detected through cpDNA markers exhibited higher levels of genetic differentiation among populations (*G*_ST_ = 0.73) than through microsatellite markers (*G*_ST_ = 0.26). Larger values for r, the ratio of migration between biparental (microsatellites) and maternally inherited markers (Ennos, 1994; Petit et al., 2005), indicate that gene flow through pollen is quantitatively much more important than seeds. For *O. alpina*,* r* = 5.62, which is smaller than the median value of 17 for 93 angiosperms species (Petit et al., 2005). This lower value for the pollen/seed migration ratio is due to the relatively high *G*_ST_ value, which is necessary for calculating *r* (see Petit et al., 2005 equation in Section 2). When calculated from nuclear microsatellite data (pollen flow), the *G*_ST_ value for *O. alpina* is relatively large in comparison with the overall value found for angiosperms (*G*_ST_ = 0.73 for *O. alpina* vs. average *G*_ST_ = 0.63 for angiosperms). Therefore, gene flow via pollen and thus the mp/ms ratio (pollen and seed gene flow ratio) are smaller for *O. alpina* than the average found for angiosperms.

The current distribution of *O. alpina* at the tops of isolated mountains combined with historical fragmentation events in the past has led to the isolation and divergence among populations, producing high levels of genetic structure that have been detected through pollen and seed genetic markers.

We also found differences in the pattern of genetic structure between pollen and seeds in *O. alpina*. For example, the chloroplast markers showed a strong association between genetic and geographic distances. Five genetically differentiated groups with a strong latitudinal component were revealed through cpDNA markers (Pérez‐Alquicira et al., 2010). Aside from the possibility of homoplasy in microsatellite markers, these divergent patterns in phylogeographic structure between nuclear and cpDNA could be explained by differences in the patterns of gene flow through pollen and seeds because pollen movement in *O. alpina* is mainly influenced by insects, particularly bees, while the dispersion of seeds is achieved by ballistic dispersal of seeds from the capsule (S.G. Weller, personal observation). Therefore, gene flow should be higher for nuclear markers carried by pollen than for organellar markers carried by seeds and may explain the detection of more genetically differentiated groups of populations using cpDNA than nuclear markers. Divergent patterns of genetic structure might also be explained by the more rapid coalescence of uniparentally inherited haploid alleles in mitochondrial or chloroplast DNA that have smaller effective population sizes than most nuclear loci (Birky et al., 1989; Palumbi et al., 2001). Smaller effective population size for organellar DNA accelerates the processes of genetic drift in neutral markers, producing more rapid genetic divergence among populations (Palumbi et al., 2001). These processes may in part explain the strong subdivision observed through cpDNA in *O. alpina*, where five genetic groups of populations were detected.

One of the main results from the popgraph analyses is that the six distylous populations included in this study were assigned to two subgroups and were not all directly connected by gene flow. This is also supported by the results from the Bayesian assignment, neighbor‐joining phenogram, and PCoA. According to the population network, three populations (two distylous and one tristylous) from the Chiricahua Mts in cluster I are genetically similar, which is also supported by the results from the Structure and NJ phenogram. These three populations are also located in close geographic proximity to each other, representing a remarkable example of the transition from tristyly to distyly. Weller et al. (2007) found that the Chiricahua Mountains populations harbor a northern–southern gradient of mid‐styled morph frequency with some populations having reduced frequency of mid‐styled morphs, suggesting that the evolution to distyly is an ongoing process within this geographic range. Our data for this area seem to provide further support for this hypothesis. However, in the three distylous populations included in the cluster II (Sierra Ancha, Pinal, Pinaleño), distyly probably evolved independently from the transition in the Chiricahua Mts.

We did not find a significant association between genetic distances and mid‐styled morph frequency, indicating that distylous populations are genetically dissimilar, and therefore distyly probably originated from different tristylous populations. Additionally, genetic evidence from chloroplast data further suggests that distyly has evolved more than once (Pérez‐Alquicira et al., 2010). If distyly is the result of convergent evolution, we expect that deterministic evolutionary forces such as natural selection have played an important role in the evolution of distyly. Modifications of the tristylous incompatibility system support this result. For example, Weller et al. (2007) found that the loss of key elements of the heterostylous incompatibility system in long‐ and short‐styled morphs increases the degree of cross compatibility between these morphs and leads to genic selection against mid alleles. Populations with higher levels of genic selection against the midallele also have higher levels of self‐fertilization for mid‐styled morphs (Weber et al., 2013). Selfing in the mid morph will result in expression of inbreeding depression which may lead to further decline in the frequency of this morph. As these deterministic forces lead to reduced frequency of the midmorph, this morph will be increasingly sensitive to genetic drift, especially with low population sizes. We propose that once populations migrated to northward ranges as a consequence of climatic oscillations, those populations with low frequencies of mid‐styledmorph were more likely to lose this reproductive morph due to genetic drift. This process could explain partially the distribution of distylous population in northern ranges of the Sky Island region.

Overall, our results indicate that microsatellite markers provide useful genetic information for tracing the possible routes of colonization of populations of *O. alpina* in the Sky Island region. The occurrence of southern and northern populations in cluster II, together with a marginally significant reduction in genetic diversity toward northern areas (*H*_E_ parameter), support that the route of colonization was probably from south to north, although the genetic signature of northern founder events inferred from microsatellites is not as clear as from cpDNA markers (Pérez‐Alquicira et al., 2010). Niche modeling also showed that the distribution of *O. alpina* during the last glacial maximum (18,000) occurred mainly in the southern ranges compared with the current distribution (Pérez‐Alquicira et al., 2010). Northward migration has been a general pattern of colonization in section *Ionoxalis* because of the South America origin of this section, and species have dispersed on several occasions to North America (Gardner et al., 2012). In view of the reduced genetic differentiation for microsatellites compared to cpDNA genetic markers, which is characteristic of most angiosperms species (Petit et al., 2005), the detection of two genetic lineages via microsatellite markers compared to five genetic clusters via cpDNA is not surprising. Microsatellite markers revealed that distylous populations were associated with different tristylous populations, and this result is also supported by our previous findings using cpDNA suggesting that the tristyly–distyly transition occurred more than once within the Sky Island region. These results suggest that natural selection has played an important role in the evolution of the reproductive system of *O. alpina*. Moreover, because distylous populations are mainly located in northern ranges where founder events were frequent, genetic drift is likely to have influenced the evolutionary transition of tristyly–distyly system.

## CONFLICT OF INTEREST

None declared.

## AUTHORS CONTRIBUTIONS

All authors contributed to the design of the study and sample collection. JP‐A and OVT acquired sequencing and microsatellite data, respectively, and conducted data analyses. JP‐A, OVT, and SGW wrote the first draft of the manuscript, and all authors contributed critically to its revision and provided approval for the final submission.

## References

[ece34096-bib-0001] Baena‐Díaz, F. , Fornoni, J. , Sosenski, P. , Molina‐Freaner, F. E. , Weller, S. G. , Pérez‐Ishiwara, R. , & Dominguez, C. A. (2012). Changes in reciprocal herkogamy during the tristyly–distyly transition in *Oxalis alpina* increase efficiency in pollen transfer. Journal of Evolutionary Biology, 25, 574–583. https://doi.org/10.1111/j.1420-9101.2012.02455.x 22268844 10.1111/j.1420-9101.2012.02455.x

[ece34096-bib-0002] Barber, P. H. (1999). Phylogeography of the canyon treefrog, *Hyla arenicolor* (Cope) based on mitochondrial DNA sequence data. Molecular Ecology, 8, 547–562. https://doi.org/10.1046/j.1365-294x.1999.00593.x 10327656 10.1046/j.1365-294x.1999.00593.x

[ece34096-bib-0003] Barrett, S. C. H. (1992). Heterostylous genetic polymorphisms: Model systems for evolutionary analysis In Spencer S. C. H. (Ed.), Evolution and function of heterostyly (pp. 1–29). Berlin, Heidelberg: Springer.

[ece34096-bib-0004] Barrett, S. C. H. (2013). The evolution of plant reproductive systems: How often are transitions irreversible? Proceedings of the Royal Society of London. Series B. Biological Sciences, 280, 20130913 https://doi.org/10.1098/rspb.2013.0913 23825207 10.1098/rspb.2013.0913 PMC3712442

[ece34096-bib-0005] Birky, C. W. , Fuerst, P. , & Maruyama, T. (1989). Organelle gene diversity under migration, mutation, and drift: Equilibrium expectations, approach to equilibrium, effects of heteroplasmic cells, and comparison to nuclear genes. Genetics, 121, 613–627. 2714640 10.1093/genetics/121.3.613 PMC1203645

[ece34096-bib-0006] Boyd, A. (2002). Morphological analysis of Sky Island populations of *Macromeria viridiflora* (Boraginaceae). Systematic Botany, 27, 116–126.

[ece34096-bib-0007] Clark, L. V. , & Jasieniuk, M. (2011). POLYSAT: An R package for polyploidy microsatellite analysis. Molecular Ecology Resources, 11, 562–566. https://doi.org/10.1111/j.1755-0998.2011.02985.x 21481215 10.1111/j.1755-0998.2011.02985.x

[ece34096-bib-0008] Clark‐Tapia, R. , & Molina‐Freaner, F. (2003). The genetic structure of a columnar cactus with a disjunct distribution: *Stenocereus gummosus* in the Sonoran desert. Heredity, 90, 443–450. https://doi.org/10.1038/sj.hdy.6800252 12764419 10.1038/sj.hdy.6800252

[ece34096-bib-0500] Conord, C. , Gurevitch, J. , &, Fady, B. , (2012). Large‐scale longitudinal gradients of genetic diversity: a meta‐analysis across six phyla in the Mediterranean basin. Ecol. Evol., 2, 2595–2609. 10.1002/ece3.350 PMC3492785 23145344

[ece34096-bib-0009] Cuevas, E. , Arias, D. M. , Dominiguez, C. A. , Castillo, R. A. , & Molina‐Freaner, F. (2006). The genetic structure of the gynodioecious Kallstroemia grandiflora (Zygophyllaceae): The role of male sterility and colonization history. Heredity, 97, 269–274. https://doi.org/10.1038/sj.hdy.6800849 16736062 10.1038/sj.hdy.6800849

[ece34096-bib-0010] Darwin, C. (1877). The different forms of flowers on plants of the same species (pp. 1–54). New York, NY: D. Appleton and Company.

[ece34096-bib-0011] Davis, M. B. , & Shaw, R. G. (2001). Range shifts and adaptive responses to Quaternary climate change. Science, 292, 673–679. https://doi.org/10.1126/science.292.5517.673 11326089 10.1126/science.292.5517.673

[ece34096-bib-0012] De Silva, H. N. , Hall, A. J. , Rikkerink, E. , McNeilage, M. A. , & Fraser, L. G. (2005). Estimation of allele frequencies in polyploids under certain patterns of inheritance. Heredity, 95, 327–334. https://doi.org/10.1038/sj.hdy.6800728 16094298 10.1038/sj.hdy.6800728

[ece34096-bib-0013] DeWoody, J. A. , Schupp, J. , Kenefic, L. , Busch, J. , Murfitt, L. , & Keim, P. (2004). Universal method for producing ROXlabeled size standards suitable for automated genotyping. BioTechniques, 37, 348–352. 15470886 10.2144/04373BM02

[ece34096-bib-0014] Dorken, M. E. , & Barrett, S. C. H. (2004). Chloroplast haplotype variation among monoecious and dioecious populations of *Sagittaria latifolia* (Alismataceae) in eastern North America. Molecular Ecology, 13, 2699–2707. https://doi.org/10.1111/j.1365-294X.2004.02246.x 15315682 10.1111/j.1365-294X.2004.02246.x

[ece34096-bib-0015] Downie, D. A. (2004). Phylogeography in a galling insect, grape phylloxera, *Daktulosphaira vitifoliae* (Phylloxeridae) in the fragmented habitat of the Southwest SA. Journal of Biogeography, 31, 1759–1768. https://doi.org/10.1111/j.1365-2699.2004.01075.x

[ece34096-bib-0016] Doyle, J. J. , & Doyle, J. J. (1987). A rapid DNA isolation procedure for small quantities of fresh leaf tissue. Phytochemical Bulletin, 19, 11–15.

[ece34096-bib-0501] Dray, S. & A.B. Dufour (2007). The ade4 package: implementing the duality diagram for ecologists. J. stat. softw., 22: 1–20. https://doi.org/10.1016/j.tpb.2006.07.001

[ece34096-bib-0017] Dyer, R. J. (2007). The evolution of genetic topologies. Theoretical Population Biology, 71, 71–79. https://doi.org/10.1016/j.tpb.2006.07.001 16919694 10.1016/j.tpb.2006.07.001

[ece34096-bib-0018] Dyer, R. J. (2014). R package gstudio: analyses and functions related to the spatial analysis of genetic marker data. Retrieved from https://github.com/dyerlab/gstudio.git

[ece34096-bib-0019] Dyer, R. J. , & Nason, J. D. (2004). Population Graphs: The graph theoretic shape of genetic structure. Molecular Ecology, 13, 1713–1727. https://doi.org/10.1111/j.1365-294X.2004.02177.x 15189198 10.1111/j.1365-294X.2004.02177.x

[ece34096-bib-0020] Dyer, R. J. , Nason, J. D. , & Garrick, R. C. (2010). Landscape modelling of gene flow: Improved power using conditional genetic distance derived from the topology of population networks. Molecular Ecology, 19, 3746–3759. https://doi.org/10.1111/j.1365-294X.2010.04748.x 20723052 10.1111/j.1365-294X.2010.04748.x

[ece34096-bib-0021] Earl, D. , & vonHoldt, B. M. (2012). STRUCTURE HARVESTER: A website and program for visualizing STRUCTURE output and implementing the Evanno method. Conservation Genetics, 4, 359–361. https://doi.org/10.1007/s12686-011-9548-7

[ece34096-bib-0022] Ennos, R. A. (1994). Estimating the relative rates of pollen and seed migration among plant populations. Heredity, 72, 250–259. https://doi.org/10.1038/hdy.1994.35

[ece34096-bib-0023] Ersts, P. J. (2017). [Internet] Geographic distance matrix generator (version 1.2.3). American Museum of Natural History, Center for Biodiversity and Conservation. Retrieved from http://biodiversityinformatics.amnh.org/open_source/gdmg.

[ece34096-bib-0024] Esselink, G. D. , Nybom, H. , & Vosman, B. (2004). Assignment of allelic configuration in polyploids using the MAC‐PR (microsatellite DNA allele counting‐peak ratios) method. Theoretical and Applied Genetics, 109, 402–408. 15085263 10.1007/s00122-004-1645-5

[ece34096-bib-0025] Evanno, G. , Regnaut, S. , & Goudet, J. (2005). Detecting the number of clusters of individuals using the software structure: A simulation study. Molecular Ecology, 14, 2611–2620. https://doi.org/10.1111/j.1365-294X.2005.02553.x 15969739 10.1111/j.1365-294X.2005.02553.x

[ece34096-bib-0026] Felsenstein, J. (1989). PHYLIP‐ phylogeny inference package, version. 3. 2. Cladistics, 5, 164–166.

[ece34096-bib-0027] Gardner, A. G. , Vaio, M. , Guerra, M. , & Emshwiller, E. (2012). Diversification of the American bulb‐bearing Oxalis (Oxalidaceae): Dispersal to North America and modification of the tristylous breeding system. The American Journal of Botany, 99, 152–164. https://doi.org/10.3732/ajb.1100152 22186183 10.3732/ajb.1100152

[ece34096-bib-0028] Hardy, O. J. , Charbonnel, N. , Fréville, H. , & Heuertz, M. (2003). Microsatellite allele sizes: A simple test to assess their significance on genetic differentiation. Genetics, 163, 1467–1482. 12702690 10.1093/genetics/163.4.1467 PMC1462522

[ece34096-bib-0029] Hardy, O. J. , & Vekemans, X. (2002). SPAGeDi: A versatile computer program to analyse spatial genetic structure at the individual or population levels. Molecular Ecology Notes, 2, 618–620. https://doi.org/10.1046/j.1471-8286.2002.00305.x

[ece34096-bib-0030] Hewitt, G. M. (2000). The genetic legacy of the Quaternary ice ages. Nature, 405, 907–913. https://doi.org/10.1038/35016000 10879524 10.1038/35016000

[ece34096-bib-0031] Hewitt, G. M. (2004). Genetic consequences of climatic oscillations in the Quaternary. Philosophical Transactions of the Royal Society B: Biological Sciences, 359, 183–195. https://doi.org/10.1098/rstb.2003.1388 10.1098/rstb.2003.1388 PMC1693318 15101575

[ece34096-bib-0032] Hodgins, K. A. , & Barrett, S. C. H. (2007). Population structure and genetic diversity in tristylous *Narcissus triandrus*: Insights from microsatellite and chloroplast DNA variation. Molecular Ecology, 16, 2317–2332. https://doi.org/10.1111/j.1365-294X.2007.03314.x 17561893 10.1111/j.1365-294X.2007.03314.x

[ece34096-bib-0033] Mantel, N. (1967). The detection of disease clustering and generalized regression approach. Cancer Research, 27, 209–220. 6018555

[ece34096-bib-0034] Masta, S. (2000). Phylogeography of the jumping spider *Habronattus pugillis* (Araneae: salticidae): Recent vicariance of sky islands populations? Evolution, 54, 1699–1711. https://doi.org/10.1111/j.0014-3820.2000.tb00714.x 11108597 10.1111/j.0014-3820.2000.tb00714.x

[ece34096-bib-0035] Meirmans, P. G. , & Van Tienderen, P. H. (2004). GENOTYPE and GENODIVE: Two programs for the analysis of genetic diversity of asexual organisms. Molecular Ecology Notes, 4, 792–794. https://doi.org/10.1111/j.1471-8286.2004.00770.x

[ece34096-bib-0036] Metcalfe, S. E. (2006). Late quaternary environments of the northern deserts and central transvolcanic belt of Mexico. Annals of the Missouri Botanical Garden, 93, 258–273. https://doi.org/10.3417/0026-6493(2006)93[258:LQEOTN]2.0.CO;2

[ece34096-bib-0037] Navascués, M. , & Emerson, B. C. (2005). Chloroplast microsatellites: Measures of genetic diversity and the effect of homoplasy. Molecular Ecology, 14, 1333–1341. https://doi.org/10.1111/j.1365-294X.2005.02504.x 15813774 10.1111/j.1365-294X.2005.02504.x

[ece34096-bib-0038] Nei, M. (1972). Genetic distance between populations. The American Naturalist, 106, 283–292. https://doi.org/10.1086/282771

[ece34096-bib-0039] Ober, K. A. , & Cannolly, C. T. (2015). Geometric morphometric and phylogenetic analyses of Arizona Sky Island populations of *Scaphinotus petersi* Roeschke (Coleoptera: Carabidae). Zoological Journal of the Linnean Society, 175, 107–118. https://doi.org/10.1111/zoj.12269

[ece34096-bib-0040] Ober, K. A. , Matthews, B. , Ferrieri, A. , & Kuhn, S. (2011). The evolution and age of populations of *Scaphinotus petersi* Roeschke on Arizona Sky Islands (Coleoptera, Carabidae, Cychrini). ZooKeys, 147, 183–197. https://doi.org/10.3897/zookeys.147.2024 10.3897/zookeys.147.2024 PMC3286259 22371665

[ece34096-bib-0041] Oddou‐Muratorio, S. , Vendramin, G. G. , Buiteveld, J. , & Fady, B. (2009). Population estimators or progeny tests: What is the best method to assess null allele frequencies at SSR loci? Conservation Genetics, 10, 1343–1347. https://doi.org/10.1007/s10592-008-9648-4

[ece34096-bib-0042] Palumbi, S. R. , Cipriano, F. , & Hare, M. P. (2001). Predicting nuclear gene coalescence from mitochondrial data: The three‐times rule. Evolution, 55, 859–868. https://doi.org/10.1554/0014-3820(2001)055[0859:PNGCFM]2.0.CO;2 11430646 10.1554/0014-3820(2001)055[0859:pngcfm]2.0.co;2

[ece34096-bib-0043] Pérez‐Alquicira, J. , Molina‐Freaner, F. E. , Piñero, D. , Weller, S. , Martínez‐Meyer, E. , Rozas, J. , & Domínguez, C. A. (2010). The role of historical factors and natural selection in the evolution of breeding Systems of *Oxalis alpina* in the Sonoran desert “Sky islands”. Journal of Evolutionary Biology, 23, 2163–2175. https://doi.org/10.1111/j.1420-9101.2010.02075.x 20840309 10.1111/j.1420-9101.2010.02075.x

[ece34096-bib-0044] Petit, R. J. , Aguinagalde, I. , de Beaulieu, J. L. , Bittkau, C. , Brewer, S. , Cheddadi, R. , … Mohanty, A. (2003). Glacial refugia: Hotspots but not melting pots of genetic diversity. Science, 300, 1563–1565. https://doi.org/10.1126/science.1083264 12791991 10.1126/science.1083264

[ece34096-bib-0045] Petit, R. J. , Duminil, J. , Fineschi, S. , Hampe, A. , Salvini, D. , & Vendramin, G. G. (2005). Comparative organization of chloroplast, mitochondrial and nuclear diversity in plant populations. Molecular Ecology, 14, 689–701. 15723661 10.1111/j.1365-294X.2004.02410.x

[ece34096-bib-0046] Petit, R. J. , Kremer, A. , & Wagner, D. B. (1993). Finite island model for organelle and nuclear genes in plants. Heredity, 71, 630–641. https://doi.org/10.1038/hdy.1993.188

[ece34096-bib-0047] Pritchard, J. K. , Stephens, M. , & Donnelly, P. (2000). Inference of population structure from multilocus genotype data. Genetics, 155, 945–959. 10835412 10.1093/genetics/155.2.945 PMC1461096

[ece34096-bib-0048] R Development Core Team (2013). R: A language and environment for statistical computing. Vienna, Austria: R Foundation for Statistical Computing Retrieved from http://www.Rproject.org

[ece34096-bib-0049] RStudio Team (2015). RStudio: Integrated development for R. Boston, MA: RStudio, Inc Retrieved from http://www.rstudio.com/

[ece34096-bib-0050] Schmitt, T. (2007). Molecular biogeography of Europe: Pleistocene cycles and postglacial trends. Frontiers in Zoology, 4, 11 https://doi.org/10.1186/1742-9994-4-11 17439649 10.1186/1742-9994-4-11 PMC1868914

[ece34096-bib-0051] Sears, B. B. (1980). Elimination of plastids during spermatogenesis and fertilization in the plant kingdom. Plasmid, 4, 233–255. https://doi.org/10.1016/0147-619X(80)90063-3 7012867 10.1016/0147-619x(80)90063-3

[ece34096-bib-0052] Silva‐Montellano, A. , & Eguiarte, L. E. (2002). Geographic patterns in the reproductive ecology of Agave lechuguilla (Agavaceae) in the Chihuahuan desert. II. Genetic variation, differentiation, and inbreeding estimates. The American Journal of Botany, 90, 700–706. 10.3732/ajb.90.5.700 21659165

[ece34096-bib-0053] Slatkin, M. (1995). A measure of population subdivision based on microsatellite allele frequencies. Genetics, 139, 457–462. 7705646 10.1093/genetics/139.1.457 PMC1206343

[ece34096-bib-0054] Sosenski, P. , Fornoni, J. , Molina‐Freaner, F. E. , Weller, S. G. , & Dominguez, C. A. (2010). Changes in sexual organ reciprocity and phenotypic floral integration during the tristyly‐distyly transitions in *Oxalis alpina* . New Phytologist, 185, 829–840. https://doi.org/10.1111/j.1469-8137.2009.03105.x 19968800 10.1111/j.1469-8137.2009.03105.x

[ece34096-bib-0055] Stockwell, D. R. B. , & Peters, D. (1999). The GARP modelling system: Problems and solutions to automated spatial prediction. International Journal of Geographical Information Science, 13, 143–158. https://doi.org/10.1080/136588199241391

[ece34096-bib-0056] Szpiech, Z. A. , Jakobsson, M. , & Rosenberg, N. A. (2008). ADZE: A rarefaction approach for counting alleles private to combinations of populations. Bioinformatics, 24, 2498–2504. https://doi.org/10.1093/bioinformatics/btn478 18779233 10.1093/bioinformatics/btn478 PMC2732282

[ece34096-bib-0057] Tsyusko, O. V. , Tuberville, T. D. , Peters, M. B. , Craword, N. , Hagen, C. , Weller, S. G. , … Glenn, T. (2007). Microsatellite markers isolated from polyploid wood‐sorrel *Oxalis alpina* (Oxalidaceae). Molecular Ecology Notes, 7, 1284–1286. https://doi.org/10.1111/j.1471-8286.2007.01856.x

[ece34096-bib-0058] Weber, J. J. , Weller, S. G. , Sakai, A. K. , Tsyusko, O. V. , Glenn, T. C. , Domínguez, C. A. , … Nguyen, K. (2013). The role of inbreeding depression and mating system in the evolution of heterostyly. Evolution, 67, 2309–2322. https://doi.org/10.1111/evo.12123 23888853 10.1111/evo.12123

[ece34096-bib-0059] Weller, S. G. (1981). Pollination biology of heteromorphic populations of *Oxalis alpina* in southeastern Arizona. Botanical Journal of the Linnean Society, 4, 57–71.

[ece34096-bib-0060] Weller, S. G. , & Denton, M. F. (1976). Cytogeographic evidence for the evolution of distyly from tristyly in the North American species of *Oxalis* section *Ionoxalis* . The American Journal of Botany, 63, 120–125. https://doi.org/10.1002/j.1537-2197.1976.tb11791.x

[ece34096-bib-0061] Weller, S. G. , Dominguez, C. A. , Molina‐Freaner, F. E. , Fornoni, J. , & LeBuhn, G. (2007). The evolution of distyly from tristyly in populations of *Oxalis alpina* (Oxalidaceae) in the Sky Islands of the Sonoran desert. The American Journal of Botany, 94, 972–985. https://doi.org/10.3732/ajb.94.6.972 21636466 10.3732/ajb.94.6.972

[ece34096-bib-0062] Zhou, W. , Barrett, S. C. H. , Wang, H. , & Li, D.‐Z. (2012). Loss of floral polymorphism in heterostylous *Luculia pinceana* (Rubiaceae): A molecular phylogeographic perspective. Molecular Ecology, 21, 4631–4645. https://doi.org/10.1111/j.1365-294X.2012.05707.x 22970974 10.1111/j.1365-294X.2012.05707.x

